# Collagen Promotes Higher Adhesion, Survival and Proliferation of Mesenchymal Stem Cells

**DOI:** 10.1371/journal.pone.0145068

**Published:** 2015-12-14

**Authors:** Chinnapaka Somaiah, Atul Kumar, Darilang Mawrie, Amit Sharma, Suraj Dasharath Patil, Jina Bhattacharyya, Rajaram Swaminathan, Bithiah Grace Jaganathan

**Affiliations:** 1 Stem Cell Biology Lab, Department of Biosciences and Bioengineering, Indian Institute of Technology Guwahati, Guwahati, Assam, India; 2 Department of Biosciences and Bioengineering, Indian Institute of Technology Guwahati, Guwahati, Assam, India; 3 Department of Hematology, Gauhati Medical College Hospital, Assam, India; Rutgers - New Jersey Medical School, UNITED STATES

## Abstract

Mesenchymal stem cells (MSC) can differentiate into several cell types and are desirable candidates for cell therapy and tissue engineering. However, due to poor cell survival, proliferation and differentiation in the patient, the therapy outcomes have not been satisfactory. Although several studies have been done to understand the conditions that promote proliferation, differentiation and migration of MSC *in vitro* and *in vivo*, still there is no clear understanding on the effect of non-cellular bio molecules. Of the many factors that influence the cell behavior, the immediate cell microenvironment plays a major role. In this context, we studied the effect of extracellular matrix (ECM) proteins in controlling cell survival, proliferation, migration and directed MSC differentiation. We found that collagen promoted cell proliferation, cell survival under stress and promoted high cell adhesion to the cell culture surface. Increased osteogenic differentiation accompanied by high active RHOA (Ras homology gene family member A) levels was exhibited by MSC cultured on collagen. In conclusion, our study shows that collagen will be a suitable matrix for large scale production of MSC with high survival rate and to obtain high osteogenic differentiation for therapy.

## Introduction

Mesenchymal stem cells differentiate into diverse cell types including osteocytes, adipocytes, chondrocytes, neurons, cardiomyocytes and endothelial cells which make them good candidates for use in regenerative therapy [1–4]. Several cues from the cellular and non-cellular microenvironment of the cells guide their maintenance in the stem cell-like state, proliferation, differentiation or migration [5, 6]. Obtaining sufficient number of cells to initiate cell therapy is a limiting factor during therapeutic applications. Culturing the cells long-term *in vitro* to obtain sufficient cell numbers might alter the gene regulation as well as the differentiation potential of these cells due to exposure to long-term cell culture induced stress. Furthermore, cell death after *in vivo* injection of MSC is also a limiting factor as majority of donor MSC are cleared after injection and they do not engraft in large numbers in the recipient system [7]. So, this implies that a high number of cells have to be injected to obtain the desired effect *in vivo*. This necessitates a better cell expansion system that promotes high cell proliferation, cell survival and differentiation. By modifying the non-cellular microenvironment, the cells can be directed to proliferate, survive or differentiate. Several studies on the non-cellular microenvironment have shown that cell shape [8–10] as well as matrix topology [11–13] of the culture surface controlled MSC differentiation. When the cells were more spread with higher actin polymerization, they differentiated into osteocytes and MSC made to form a spherical morphology differentiated into adipocytes. Other studies have shown that matrix rigidity [14] combined with the mechanical feedback provided by the extracellular matrix proteins guided MSC differentiation [15, 16]. A recent study reported that MSC received biochemical signals for differentiation through early extracellular matrix protein before the extracellular matrix protein were modified by the cells during differentiation [17]. All these studies strongly suggest that non-cellular physical microenvironment regulates MSC differentiation which can be modified for directed cell differentiation during tissue engineering.

Previous studies have tested the effect of different ECM substances collagen and fibronectin on MSC differentiation for coating on scaffold or as injectable biomaterial for tissue repair. Coating the cell culture surface with collagen alone [18–20] or in combination with other scaffold material induced osteogenic differentiation of MSC [21–25] in some cases, in the absence of osteo-inductive factors. Also, MSC cultured on fibronectin coated plates had enhanced osteogenic differentiation [26–28].

However, multiple factors come in to play when utilizing MSC for therapy. Firstly, a rapid expansion of MSC *in vitro* is required prior to utilizing the cells for injection into the patient. While expanding the cells, it is necessary that the cells maintain their self-renewal and multipotent differentiation capacity. Secondly, when the cells are administered with a scaffold for therapy, a suitable matrix that provides cell migration for tissue regeneration, cell attachment and survival during stress conditions is necessary. In this context, we performed a systematic analysis of various properties of MSC cultured on collagen and fibronectin as well as commonly used cell adhesion factor poly-L-lysine for their potential use in cell therapy for *in vitro* expansion of cells or for coating in scaffolds to improve their therapeutic potential.

## Materials and Methods

The current study is approved and ethical clearance provided by Institute Human Ethics Committee (IHEC) of Indian Institute of Technology Guwahati (IITG).

### Bone marrow mesenchymal stem cells

Bone marrow aspirates were obtained from iliac crest of patients referred to Department of Hematology, Gauhati Medical College Hospital (GMCH) after written informed consent as per GMCH ethical committee guidelines. The bone marrow cells were subjected to red cell lysis using ammonium chloride solution (0.15M, pH 7.3) and plated in media containing 10% FBS at a density of 1x10^5^ cells/cm^2^. The non-adherent cells were removed after 48 hours and colonies containing spindle shaped cells appeared after 2–3 weeks in culture. The isolated MSC were positive for the cell surface markers CD13, CD44, CD73, CD90, CD105 and HLA class I and negative for CD34 and CD45. The MSC used in the experiments were from passage 2–5 and wherever late passage cells were required, the cells were used at passage 10–12.

### ECM coating

The tissue culture treated plates/flasks (BD biosciences) were coated with collagen type I (from calf skin), fibronectin (from bovine plasma) or poly-l-lysine. The required concentration of collagen (2ug/cm^2^), poly-l-lysine (100ng/cm^2^) or fibronectin (100ng/cm^2^) [29–31] was diluted in PBS and tissue culture plates were coated at 37°C for 1hr. The unbound substrate was washed with PBS and the plates were used either immediately or stored at 4°C for 24-48hr before use.

### Cell viability assay

MTT (3-(4,5-Dimethylthiazol-2-yl)-2,5-Diphenyltetrazolium Bromide) assay was performed as per the manufacturer’s instructions (Himedia Laboratories) to check the cell viability. Cells were seeded in a 96-well plate at a density of 500 cells/well. MTT reagent was added to the cells and incubated for 4 hours at 37°C. The resulting formazan precipitate was solubilized with the solubilization reagent and absorbance was measured at 570nm. Each sample was analysed in triplicates and average value was taken for plotting the graph.

### Adipogenic and osteogenic differentiation

MSC were differentiated into adipocytes and osteocytes as reported earlier [32]. Osteogenic differentiation was induced by addition of β-glycerolphosphate (10mM), dexamethasone (100nM) and ascorbic acid 2-phosphate (50μM) for 21–35 days in DMEM containing 10% FBS and percentage differentiation was analysed by staining for alkaline phosphatase and calcium deposition was determined by Alizarin red staining. Quantification was done by eluting Alizarin red with cetylpyridinium chloride and absorbance measurement at 562nm.

Adipogenic differentiation was carried out in DMEM with 10% FBS supplemented with dexamethasone (1μM), indomethacin (200μM), iso butyl methyl xanthine (500μM) and insulin (10mM) for 21–30 days and differentiation was analysed by staining with oil-red O. Oil Red O positive cells were counted microscopically and the stain was extracted from the cells after counting and quantified by absorbance measurement at 500nm.

### Wound healing assay

Ten thousand cells/cm^2^ were seeded in a 12-well plate coated with different substrates and the cells were allowed to attach for 24–36 hours or until they reached confluency. A scratch was made in the cell monolayer and cell migration was observed and documented microscopically at regular intervals until the wound closed. The migration speed of the cells was calculated by measuring the distance covered by the cells at each time point. The cells were serum starved for 12 hours prior to the migration assay to negate the effect of cell proliferation during migration.

### Flow cytometry

The cell surface protein expression was analysed by flow cytometry. The cells were incubated with anti-human fluorescent conjugated antibodies against integrins CD29, CD49a, CD49b, CD49d or CD49e for 30 minutes at 4°C. The cells were washed, resuspended in buffer containing propidium iodide and analysed with FACS caliber (BD biosciences).

### RHOA activity analysis

The active level of RHOA was determined by RHOA GLISA assay (Cytoskeleton) following the manufacturer’s instructions. Briefly, ice-cold lysis buffer provided with the kit (Cytoskeleton) was added to the cell layer and cell lysate was collected by scraping. The protein concentration of the cell lysates were measured by Bradford assay. Equal amount of protein was used for analysis of active RHOA levels by GLISA.

### Real-time PCR analysis

Gene expression analysis was performed by real-time PCR. Total RNA was extracted from cells using TriZol reagent (Invitrogen). The RNA was reverse transcribed into cDNA using first strand cDNA synthesis kit (Invitrogen) and Oligo dT primers. The primers used for real-time PCR were obtained from an earlier published report [33]. Real-time PCR was performed using Power SyBr Green reagents (Invitrogen) in an ABI 7500 (ABI) real-time PCR machine. The gene expression levels in each sample were normalized to their respective GAPDH expression levels. The fold change in the expression levels compared to the control is calculated using 2^−ΔΔCt^ method.

### Actin staining

F-actin was visualized by staining with TRITC conjugated phalloidin. Briefly, the cells were fixed with paraformaldehyde (4% for 20 minutes), permeabilised with Triton x-100 (0.1% for 15 minutes) and stained with phalloidin-TRITC. Nucleus was stained with DAPI and the cells were documented using Zeiss Axio observer and CCD camera (Zeiss).

### Total internal reflection microscopy imaging

MSC were cultured on glass coverslips (1.5 mm thickness) coated with different substrates. The cells were stained with phalloidin-TRITC to visualize F-actin which was excited with 488nm laser. OTIR (Objective type-Total internal reflection) fluorescence microscopic analysis was performed as reported earlier [34] to identify the cell-surface contact points. The fluorescence signal was collected through a long-working distance water immersion objective (63x) using a camera-based detection system (Zeiss).

### Cell proliferation analysis

Cell cycle analysis was done by staining the DNA of the cells with propidium iodide. Briefly, the cells were harvested by trypsinization, washed and fixed with ice-cold ethanol (70%) for 30 minutes at 4°C. The cells were washed, treated with RNAse A, stained with propidium iodide and cell cycle was analysed by flow cytometery.

### Cell adhesion assay

Cells were seeded on control surface (tissue culture treated), collagen, fibronectin or poly-l-lysine coated surface and allowed to adhere either for 2hr or 12hr. The non-adherent cells were gently removed by washing with PBS and adherent cells in each condition was counted.

### H_2_O_2_ treatment

MSC cultured on different surface were treated with H_2_O_2_ (400μM) [35] in DMEM containing 0.1% serum for 2–3 hours. The cells were collected by trypsinization and the cell death percentage was analysed by counting both dead and live cells using trypan blue staining. For obtaining the complete cell death percentage, cells in the supernatant were also included in the cell counting after H_2_O_2_ treatment.

### Mitochondrial staining

To visualize active mitochondria inside the cell, Tetramethylrhodamine, ethyl ester (TMRE, 100nM) [36] was added to the culture media and incubated at 37°C for 30 minutes. The cells were washed, fresh media added and mitochondrial distribution was visualized using Zeiss Axio Observer Z1 inverted microscope (Zeiss, Goettingen, Germany). TMRE was used in our study for mitochondrial staining instead of commonly used mitotracker dyes since we were interested in visualizing only the active mitochondria in the cells. An earlier study has shown that TMRE is retained only by active mitochondria with intact mitochondrial membrane potential unlike mitotracker dyes [37].

### Superoxide production analysis

Superoxide production in the cells cultured on different substrates was determined using Mitosox red mitochondrial superoxide indicator kit (Life Technologies) according to manufacturer’s instructions. The MitoSOX reagent was added to the cells and incubated at 37°C for 10 minutes. The cells were trypsinized and washed with warm buffer and MitoSOX fluorescence was analysed by flow cytometry.

### Data analysis

The flow cytometric data was analysed using flow-Jo software (Tree Star Inc). Cell migration in the wound healing assay was analysed by TScratch software. Cell-surface contact points of MSC on different coatings were analysed using ImageJ software. Statistical analysis was performed with SPSS software and values of p<0.05 were considered statistically significant. The effect of different coating on MSC proliferation, survival, migration and gene expression was analysed using paired samples T-test.

## Results

Obtaining sufficient number of MSC in a short period of time for administration into the patient following injury is highly desirable for faster recovery. Usually, MSC are isolated and expanded *in vitro* before being given to the patient. Cell culture matrix plays an important role in determining the cell behavior and we have analyzed the properties of bone marrow derived MSC cultured on matrices such as collagen (COL), fibronectin (FBN) which are components of extracellular matrix proteins and cell attachment factor poly-L-lysine (PLL).

### Cell proliferation and survival

**Fig 1:**
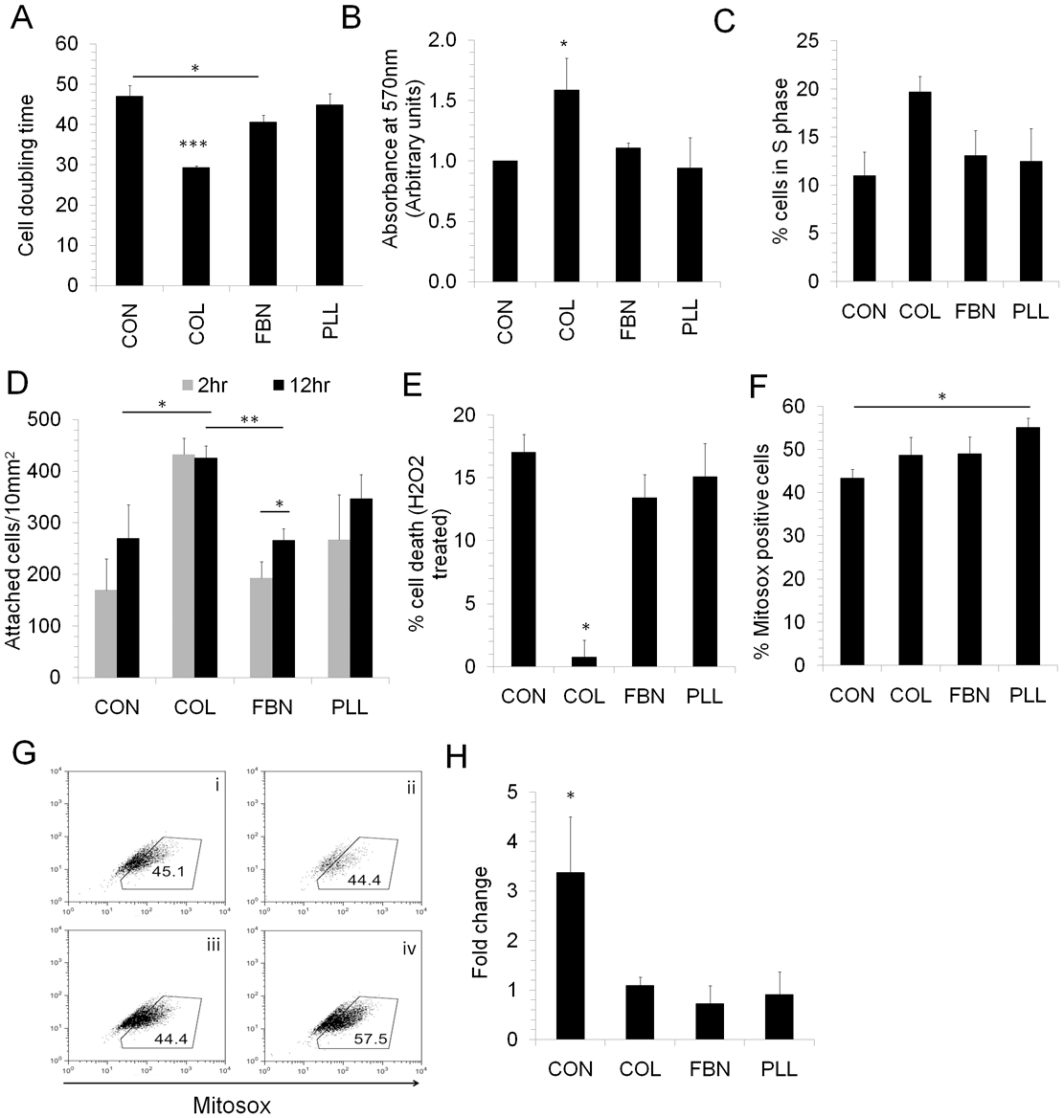
Proliferation and cell survival. MSC were seeded in equal numbers (1000 cells/cm^2^) on tissue culture dishes which were left uncoated (CON) or coated with collagen (COL, 2ug/cm2), fibronectin (FBN (100ng/cm2) or Poly-l-lysine (PLL, 100ng/cm2) for 48–72 hours. (A) Doubling time was calculated by cell counting of MSC grown on different matrices. (B) Cell proliferation on different matrices were analysed by MTT assay. *p<0.05 represents cell proliferation significantly higher in cells grown on COL compared to CON, FBN and PLL surfaces. (C) MSC cultured on COL, FBN and PLL and TC surface (CON) was collected by trypsinization and stained with propidium iodide and the cell cycle profile was analysed flow cytometrically. Values are Mean±SD, n = 5–7 samples. (D) Equal numbers of MSC were seeded on TC (CON), COL, FBN and PLL surfaces and non-adherent cells were removed after 2hr or 12hr and the adherent cells were counted. Five different high power fields were counted microscopically for each sample. Values are Mean±SD, n = 3 samples (E) MSC were treated with H_2_O_2_ (400μm) for 2–3 hours under low serum conditions (0.1% FBS) and the percentage of cell death was analysed by counting the live and dead cells in each condition. *p<0.05 represents that cell death in cells grown on COL was significantly lower compared to other conditions. (F, G) MSC grown on different matrices were serum starved for 48 hours and mitosox superoxide indicator was added to cells for 30 minutes and mitosox red fluorescence was analysed by flow cytometry and the percentage positive cells were determined. (G) Representative flow cytometric plot showing mitosox fluorescence analysis and gating in control (i), COL (ii), FBN (iii), PLL(iv). Values are Mean±SD, n = 3 independent experiments. (H) Total RNA was extracted from 5 samples cultured on uncoated TC (CON), COL, FBN and PLL coated tissue culture dishes. Semi-quantitative real-time PCR was done to analyse the mRNA expression levels of IL6 and the values were normalized to GAPDH expression levels in the respective samples. *p<0.05 represents that IL6 levels were significantly higher in control (CON) compared to other conditions. Values are Mean±SD, n = 5 samples. *p<0.05, **p<0.005, ***p<0.0005

In order to identify a suitable matrix that promotes high cell proliferation, MSC were cultured on COL, FBN, PLL and tissue culture treated plastic (TC) surfaces. High cell proliferation and decreased doubling time was seen in MSC cultured on COL whereas the doubling time was significantly higher in cells cultured on TC surface (Fig 1A and 1B). Increased proliferation on COL was further evidenced by high percentage of COL cultured MSC in S phase compared to other matrices (Fig 1C). To check the surface which promotes faster and higher cell adhesion, cell attachment on different matrices was determined after 2hr and 12hr of cell seeding. We found a significantly higher cell adhesion on COL matrix compared to TC and FBN surfaces. In case of COL, PLL and control TC surface, the cells attached within 2hr of cell seeding and no further increase in cell adhesion was observed until 12hr. However, on FBN surface, the attached cells percentage increased significantly on prolonged time period with more cells found attached at 12hr compared to 2hr (Fig 1D).

In addition to cell proliferation, cell survival is an important factor. Cells *in vivo* are subjected to oxidative as well as nutritional stress and we determined the effect of different matrices on the cell survival under stress conditions. MSC grown on different matrices were subjected to oxidative stress by H_2_O_2_ treatment under serum starvation conditions and their cell death rate was determined. While cell proliferation was negligible during serum starvation, cell death was significantly lower in cells grown on COL compared to other matrices (Fig 1E). Additionally, during serum starvation, reactive oxygen species (ROS) production was observed in cells cultured on all matrices tested, but it was significantly higher in cells cultured on PLL (Fig 1F and 1G). We also hypothesized that inflammatory cytokines produced by MSC might play an important role in their *in vivo* survival. For this, we determined IL6 mRNA levels in MSC cultured on different matrices. Whereas a significantly high IL6 expression was found in cells cultured on TC, it was significantly low in cells cultured on COL, FBN or PLL (Fig 1H).

### F-actin arrangement and cell migration

**Fig 2:**
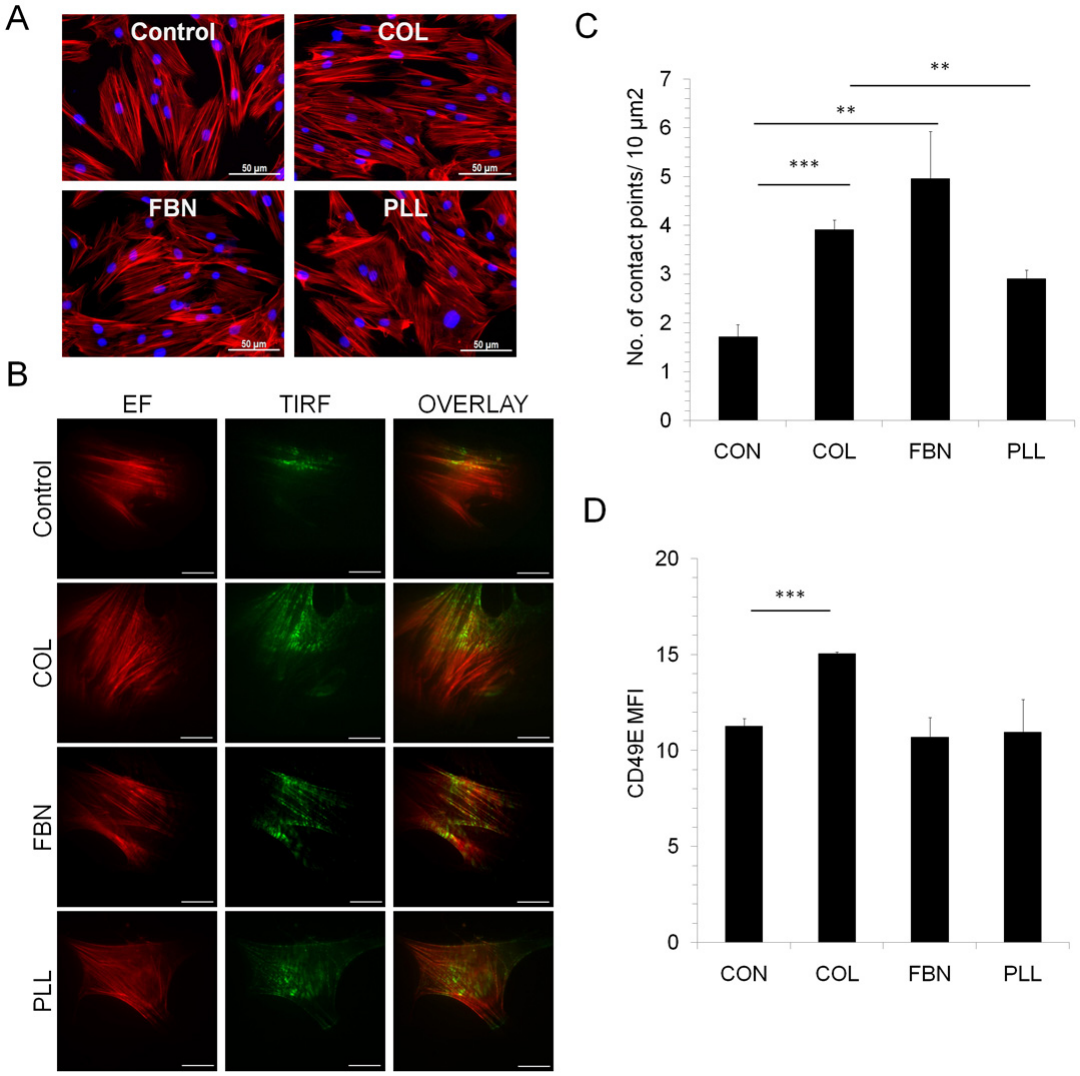
Actin cytoskeleton and cell-surface contact points of MSC. (A) MSC seeded on uncoated surface (Control), COL, FBN and PLL matrices were stained with TRITC conjugated phalloidin to visualize F-actin (red) and nucleus was stained with DAPI (blue). (B, C) The cell-contact points of MSC with different substrates was analysed by total internal reflection fluorescence microscopy (TIRF) in cells stained with phalloidin-TRITC cultured on glass coverslips. Representative images are shown; the scale bars represent 20μm. EF- Epi fluorescence. (D) The expression levels of CD49e in MSC cultured on TC surface (CON), COL, FBN and PLL surfaces were analysed by flow cytometry. The mean fluorescence intensity (MFI) for each sample was calculated based on their respective isotype control. Values are Mean±SD, n = 5 samples. **p<0.005, ***p<0.0005.

**Fig 3:**
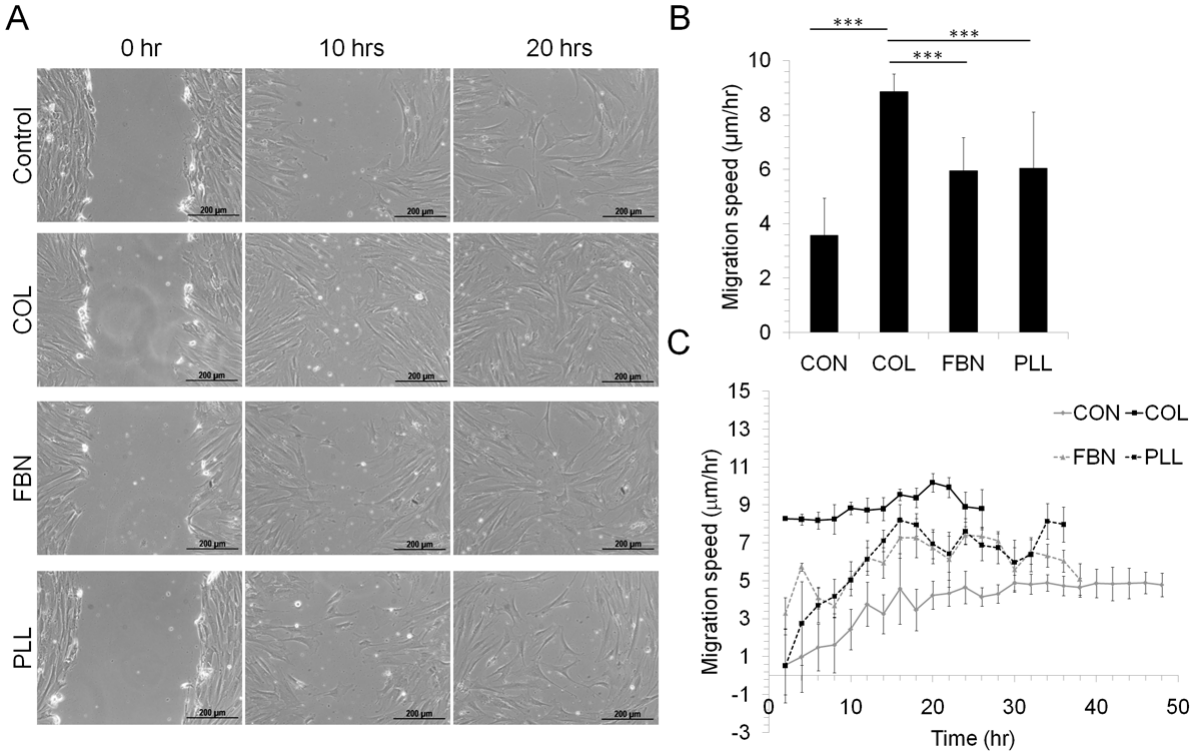
Cell migration analysis. MSC were seeded on different matrices (Control, COL, FBN, PLL) and cultured until confluence. The cells were serum starved for 24 hours to inhibit cell proliferation and a scratch was made in the confluent layer. (A) The cell migration was documented microscopically at regular time intervals (~2 hours) and the distance covered by the cells on each matrix was calculated and the average migration speed determined. (B) Average migration speed was calculated from three independent experiments and values are plotted. Values are Mean±SD, n = 3. (C) MSC migration on different matrices was determined by wound healing assay. MSC were seeded on control TC treated surface (CON), COL, FBN or PLL coated surface and the migration speed was analysed every 2 hours until the wound closed. Values are Mean+SEM, n = 3 independent experiments. ***p<0.0005.

Actin acts as mechano sensor and transduces the extracellular signals to direct cell migration, differentiation and cell shape [38]. Comparable F-actin arrangement with regular, parallel actin filaments was observed in MSC cultured on COL, FBN, PLL or control surface, however, the cells assumed a polygonal shape while proliferating on PLL and uncoated surfaces (Fig 2A). Actin also mediates the attachment of cells with the cell culture surface through integrin and other molecules. Accordingly, cell-surface contact points of MSC cultured on different matrices was determined by TIRF microscopy by staining F-actin with phalloidin-TRITC (S2 Fig). Whereas MSC showed several cell-surface contact points on COL, FBN and PLL matrices, contact points were very few on uncoated surface (Fig 2B and 2C). Integrins present on the cell surface cross-talk with the ECM molecules and intracellular signaling molecules by binding with actin filaments and regulate cell migration and differentiation. For this, expression levels of integrins CD29 (integrin beta 1), CD49D (integrin alpha 4) and CD49E (integrin alpha 5) were assessed on cells grown on different ECM. No changes were observed in the expression levels of CD29, CD49D (data not shown) whereas an increased CD49E expression was seen in cells cultured on COL (Fig 3D).

Active migration of cells in the scaffold is required for functional remodeling. For this, the migration rate of MSC on different matrices was assessed by wound healing migration assay. Cells seeded on TC, COL, FBN and PLL coated surfaces were allowed to migrate until the wound closed completely. The migration distance covered by the cells was documented microscopically at regular time intervals. Maximum migration speed was observed on COL followed by FBN and PLL with the cells on TC exhibiting the lowest migration speed. The cells grown on TC had an average migration speed of 3.5μm/hr whereas cells grown on COL showed an average migration speed of 8.8μm/hr. The cells closed the wound approximately in 24 hours on COL matrix whereas it took more than 48 hours for the cells to close the wound on TC (Fig 3A–3C). The cells were serum starved for 12 hours prior to the migration assay to negate the effect of cell proliferation in accelerating the wound healing.

### Differentiation

**Fig 4:**
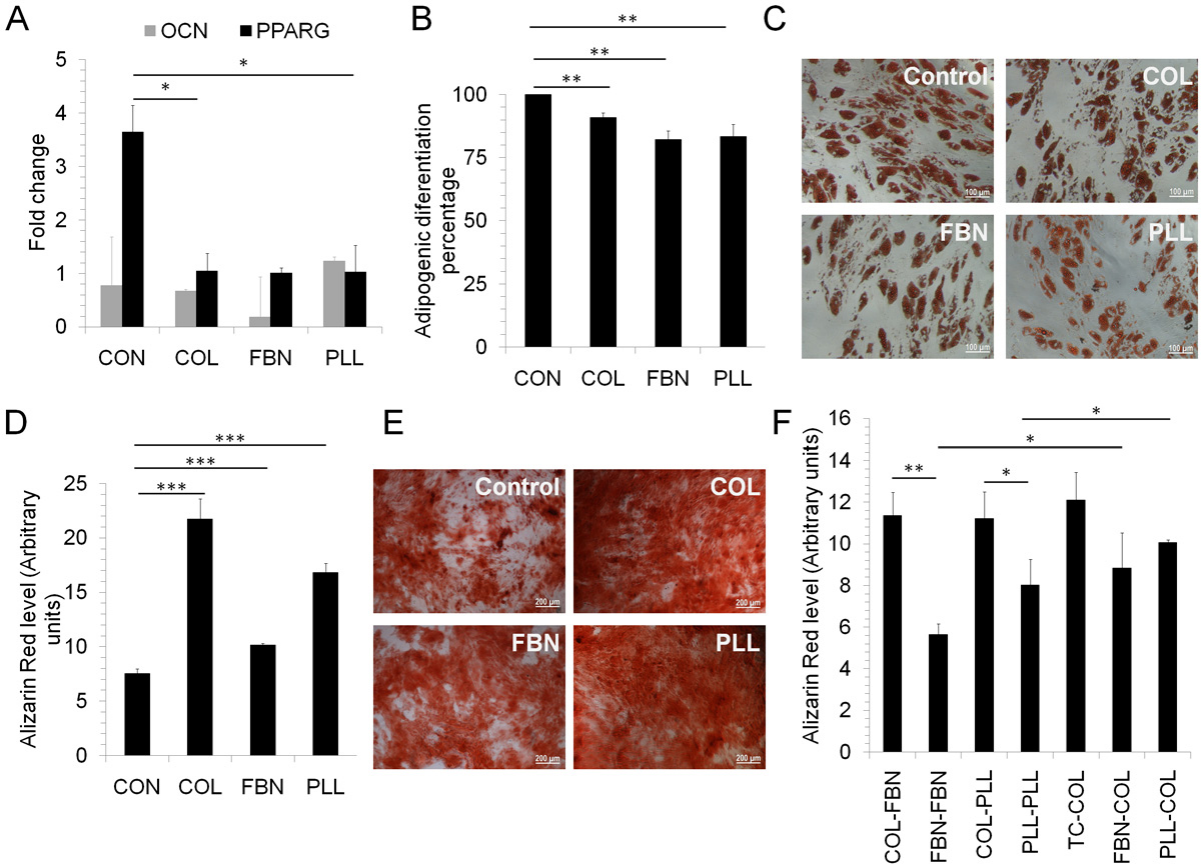
Adipogenic and osteogenic differentiation. (A) Total RNA was isolated from three MSC samples cultured on TC surface (CON) or COL, FBN or PLL coated surfaces and reverse transcribed. The mRNA expression levels of OCN and PPARG were analysed by real-time PCR and normalized to their respective GAPDH levels. Values are Mean±SE, n = 3 samples. MSC were isolated on uncoated TC surface and differentiated into either (B, C) adipocytes or (D, E) osteocytes on different matrices (Control, COL, FBN and PLL) for 21–35 days. (B, C) To detect adipogenic differentiation, the cells were stained with oil-red O and the positive cells were counted microscopically. (D, E) Osteogenic differentiation was determined by staining the cells with Alizarin Red and quantified by absorbance measurement at 562nm. Values are Mean±SD, n = 6–12. (F) Osteogenic differentiation during switching of cell culture surface. MSC were cultured for one week prior to induction of osteogenic differentiation on control TC treated surface (CON), COL, FBN or PLL. These cells were transferred to and differentiated into osteocytes either on COL, FBN or PLL surfaces and osteogenic differentiation was determined alizarin red staining. For example, COL-FBN means that the cells were cultured on COL and switched to FBN for osteogenic differentiation. Values are Mean+SD, n = 3 independent experiments.*p<0.05, **p<0.005, ***p<0.0005.

Either cells can be differentiated *in vitro* and administered into the patient or allowed to differentiate *in vivo*, an efficient directed differentiation is desirable during tissue repair. In this context, we performed gene expression analysis to check whether the matrices themselves could prime MSC into either adipogenic or osteogenic lineage. For this, the transcript levels of *OSTEOCALCIN* (OCN) and *PPAR gamma* (PPARG) was analysed in MSC cultured on COL, FBN, PLL and TC surfaces before they were induced with the differentiation media. No difference in *OCN* levels was observed in MSC cultured on different matrices, whereas a significant increase in *PPARG* expression was seen in cells grown on TC surface compared to cells cultured on other matrices (Fig 4A) suggesting a bias in the differentiation potential of TC surface expanded cells.

To check whether the cells have varied adipo- or osteo- differentiation abilities on different matrices, cells initially cultured on TC surface were differentiated into adipocytes or osteocytes on COL, FBN or PLL. In line with increased PPARG expression seen in TC surface cultured MSC, high adipogenic differentiation was observed on TC but it was lower on COL, PLL and FBN (Fig 4B and 4C). Conversely, cells on TC surface showed the least osteogenic differentiation and it was significantly high on COL surface (Fig 4D and 4E). Since COL promoted osteogenic differentiation, we tested whether cells initially expanded on COL could still have high osteogenic differentiation when differentiated on other matrices like FBN or PLL. We found a significantly high osteogenic differentiation by COL expanded cells on FBN and PLL surfaces compared to MSC expanded and differentiated on FBN or PLL (Fig 4F). Additionally, we found a significantly high osteogenic differentiation on COL surface even though the cells were expanded prior to differentiation on TC, FBN or PLL, implying that in the presence of differentiation factors, COL surface has a synergistic effect in promoting high osteogenic differentiation (Fig 4F). However, no change in adipogenic differentiation was observed during matrix switching (data not shown).

### RHOA activity

**Fig 5:**
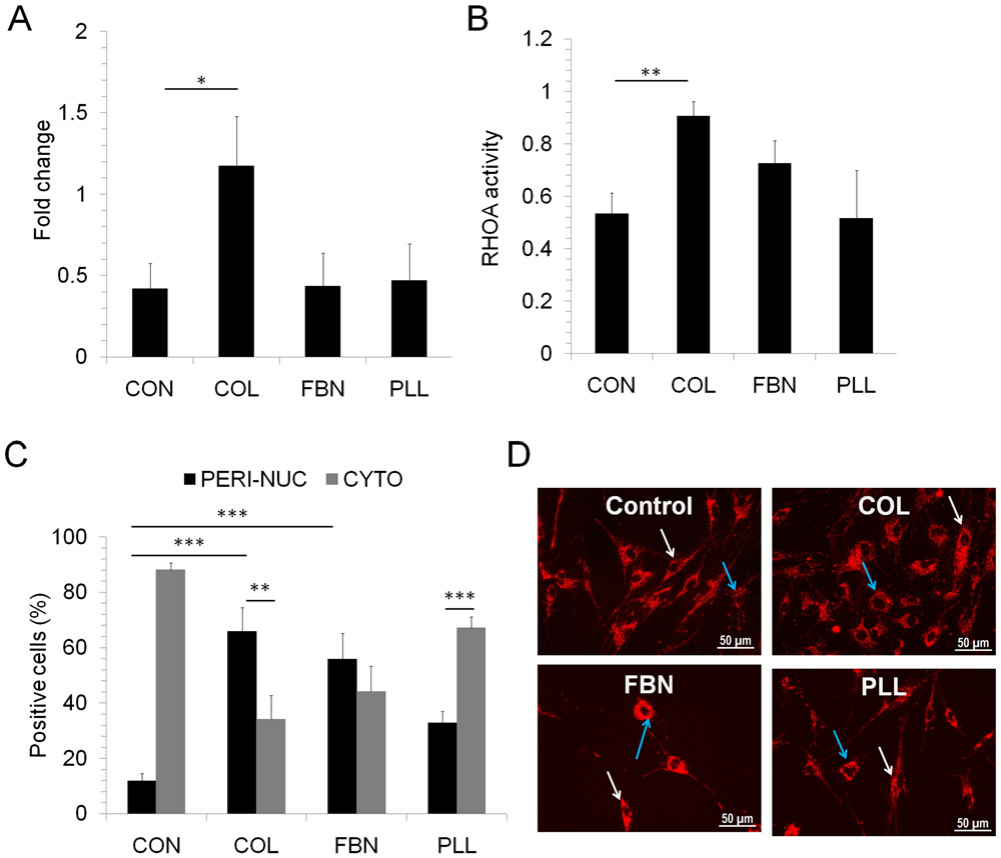
Active RHOA analysis and mitochondria distribution. (A) The RHOA mRNA levels in MSC cultured on TC surface (CON) or COL, FBN, PLL surfaces were determined by real-time PCR analysis. RHOA expression levels were normalised to GAPDH expression levels. (B) Active GTP bound RHOA levels in cells cultured on TC surface (CON), COL, FBN and PLL matrix was determined by RHOA G-LISA activation assay. Values are Mean±SD, n = 3 samples. (C, D) MSC grown on uncoated (Control, CON), COL, FBN and PLL coated surfaces were stained with TMRE to visualize the distribution of active mitochondria in the cells. (C) The percentage of cells showing cytoplasmic or peri-nuclear distribution was calculated in each condition. (D) Representative microscopic images are shown, blue arrow points to peri-nuclear staining and white arrow points to cytoplasmic staining in each condition. Values are Mean±SD, n = 6–15 (3 samples). *p<0.05, **p<0.005, ***p<0.0005.

We tested RHOA activity in MSC cultured on different matrices, since RHOA controls cell migration [32] as well as MSC differentiation [10]. Initially, RHOA expression was determined by analyzing the transcript levels by real-time PCR. MSC cultured on COL showed a significantly high RHOA mRNA level when compared to cells on other matrices (Fig 5A). However, RHOA activity is regulated at the protein level where they switch between active GTP bound state and inactive GDP bound state and downstream signaling occurs during active state [39]. The level of active RHOA, which is the GTP bound form of the protein, was determined by G-LISA and consistent with the mRNA level, high RHOA activity was seen in cells cultured on COL (Fig 5B).

### Sub-cellular mitochondrial distribution

Next, we wanted to test the mitochondrial distribution in MSC cultured on different matrices since Varum et al [40] has shown that the mitochondrial distribution could indicate the stem-like state of cells. They found that in ES cells, a peri-nuclear distribution represented a stem-cell like state and a cytoplasmic distribution represented a differentiated state [40]. The active mitochondria distribution in MSC cultured on different matrices was identified by TMRE staining. The cells cultured on COL showed predominantly peri-nuclear distribution whereas the cells grown on TC as well as PLL had high cytoplasmic mitochondrial distribution pattern (Fig 5C and 5D).

## Discussion

MSC have been used extensively for cell therapy to treat various bone related disorders with a potential to treat spinal cord injury, cardiac diseases and also for co-injection with hematopoietic cells during bone marrow transplantation to facilitate hematopoietic cell engraftment [7, 41–44]. The cell proliferation, migration, survival and differentiation during *in vitro* and *in vivo* conditions are limiting factors while utilizing these cells for cell therapy. *In vitro* cell expansion is performed prior to *in vivo* administration where a large number of cells are used (1x10^6^ cells/Kg body weight) which requires a rapid expansion system [7]. During tissue engineering applications, the cells are seeded in the matrix and injected into the site of tissue injury or seeded on solid materials to help in tissue repair [45]. Additional problem in utilizing MSC for cell therapy is their cell death *in vivo* within few hours of intravenous injection. Even after intra-osseous injection in animal models, the survival rate of bone marrow derived MSC *in vivo* was very low (unpublished observation). To address some of these issues, we studied different cell matrices that could protect MSC from oxidative/nutrient stress and promote cell growth and migration in *in vitro* and *in vivo* conditions. In the current study, we performed a detailed analysis of the cell characteristics of MSC when cultured on different cell surface matrices for their optimal growth, survival and migration.

We found that COL fibers significantly promoted cell proliferation resulting in short doubling time compared to TC surface. Additionally, COL matrix was efficient in protecting MSC from oxidative and nutrient stress induced cell death that might occur *in vivo* during ischemia [46]. The cell adhesion on COL was also high and it occurred within a short period of cell seeding (2hr) and no increase in cell adhesion was seen at 12hr except on FBN. Although high cell attachment was observed also in PLL, there was also high ROS production and high oxidative stress induced cell death on PLL surface. These results suggest that culturing MSC on COL will help us to achieve high cell proliferation and survival and COL can be used as a coating on scaffold for *in vivo* administration of MSC to protect them from stress induced cell death.

Moreover, high osteogenic differentiation was observed when MSC were differentiated on COL even when they were cultured on other matrices. Similarly, high osteogenic differentiation was retained even when the cells were differentiated on FBN or PLL if they have been cultured on COL prior to induction of differentiation. High osteogenic differentiation on COL also correlates with increased RHOA GTPase activity in COL cultured MSC where McBeath et al [10] observed higher osteogenic differentiation of MSC when active RHOA (RHOAV14) was exogenously over-expressed. These results suggest that COL is a suitable cell expansion matrix to retain high osteogenic differentiation potential of MSC and provide a synergistic effect in promoting osteogenic differentiation in the presence of induction factors prior to *in vivo* administration. Similar increase in osteogenic differentiation on COL surface was also reported by Linsley et al and others [21, 22, 24–26, 28, 47]. Although chondrogenic differentiation is an important property of MSC, it was not tested in the current study as chondrogenic differentiation *in vitro* is usually performed as a pellet culture where there was no scope for cell to cell-culture-surface contact.

Furthermore, active cell migration is essential in tissue engineering to form structurally and biologically functional system for tissue repair. COL promoted higher cell migration and cell attachment with more cell to cell surface contact points. In addition, we observed increased CD49e levels in COL cultured MSC and CD49e was shown to mediate migration of hematopoietic stem cells [48] and our earlier study showed that CD49e cell surface expression increased during osteogenesis [33]. This suggests that CD49e increase in COL cultured MSC might be associated with its osteogenic differentiation potential. Further studies on CD49e silencing or inhibition will provide more insight into the phenomenon since CD49e silencing inhibited cell adhesion in other cell types [49]. Interestingly, MSC cultured on different matrices showed substrate specific mitochondrial distribution pattern. The predominantly peri-nuclear mitochondrial distribution on COL suggests that COL matrix might maintain MSC in more stem cell like state.

Although the matrix rigidity and elasticity were not tested in our study, the results show that differential mechano-sensing and signaling was promoted in cells cultured on different matrices. Even though other studies have shown the usefulness of COL and FBN as suitable extracellular matrix for osteogenic differentiation of MSC [21, 22, 24–26, 28, 47], our study specifies their suitability in enhancing cell attachment, migration, cell survival and maintaining their self-renewal and differentiation potential. Taken together, our results suggest that COL might be employed as a matrix during *in vitro* cell expansion to maintain their osteogenic differentiation potential and coating *in vivo* scaffolds for increased cell adhesion, proliferation, survival during stress conditions and migration. Additionally, to improve the tissue regeneration and repair *in vivo*, MSC can be pre-differentiated *in vitro* and administered intravenously or on scaffold which will reduce the time required for tissue repair and regeneration.

## Conclusion

In conclusion, our study demonstrates that extracellular matrices induce differential cell behavior of MSC by altering their cell proliferation, migration and differentiation. MSC can be pre-differentiated on COL to obtain high number of osteocytes or on uncoated tissue culture treated plastic to obtain adipocytes based on the therapy requirement. Increased proliferation can be achieved by coating the cell growth surface with COL and when used in tissue engineering COL coated scaffolds will also promote high cell migration, proliferation, survival and osteogenic differentiation.

## Supporting Information

S1 Fig Cell cycle analysis of MSC. Representative flow cytometric plot showing the cell cycle analysis of MSC cultured on TC, COL, FBN and PLL.
(TIF)

S2 Fig Analysis of cell to cell surface contact points using ImageJ. The cell to cell surface contact points of MSC on cell culture surfaces coated with COL, FBN, PLL or uncoated was analysed as shown in the figure. For this MSC were stained with TRITC labeled phalloidin. Epifluorescence figures (A) were analysed to calculate the cell surface area and TIRF images (B) were processed to obtain the number of contact points.
(TIF)
