# Supplementary figures and images for: Collagen Promotes Higher Adhesion, Survival and Proliferation of Mesenchymal Stem Cells
Source: PLoS One. 2015 Dec 14;10(12):e0145068. doi: 10.1371/journal.pone.0145068 (PMC4678765; doi:10.1371/journal.pone.0145068)

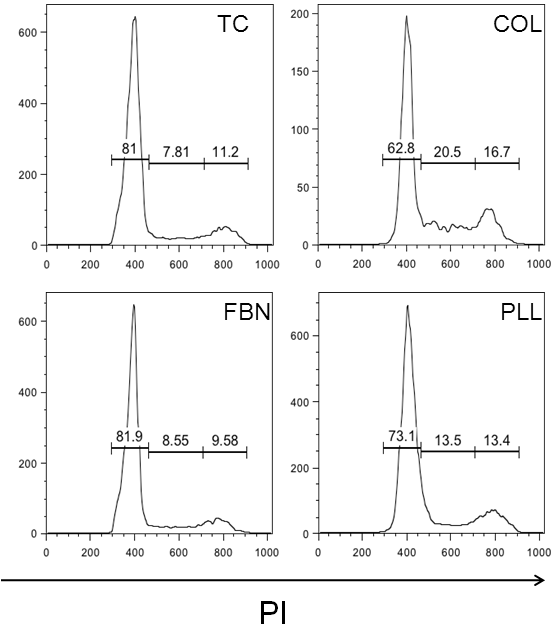

Supplement: S1 Fig — Representative flow cytometric plot showing the cell cycle analysis of MSC cultured on TC, COL, FBN and PLL. (TIF) [file pone.0145068.s001.tif]

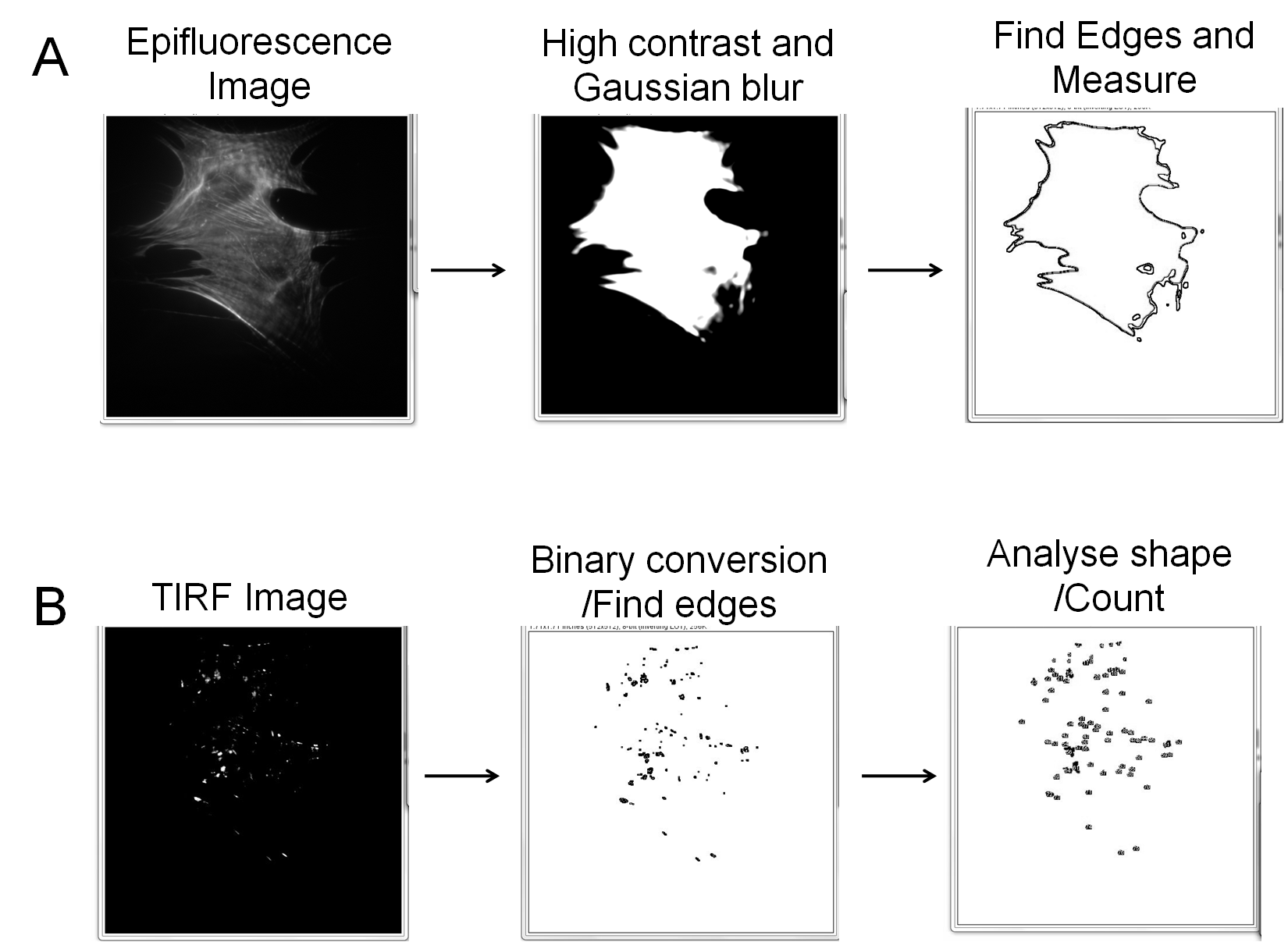

Supplement: S2 Fig — The cell to cell surface contact points of MSC on cell culture surfaces coated with COL, FBN, PLL or uncoated was analysed as shown in the figure. For this MSC were stained with TRITC labeled phalloidin. Epifluorescence figures (A) were analysed to calculate the cell surface area and TIRF images (B) were processed to obtain the number of contact points. (TIF) [file pone.0145068.s002.tif]
